# Biologically active recombinant human erythropoietin expressed in hairy root cultures and regenerated plantlets of *Nicotiana tabacum* L.

**DOI:** 10.1371/journal.pone.0182367

**Published:** 2017-08-11

**Authors:** Poornima Devi Gurusamy, Holger Schäfer, Siva Ramamoorthy, Michael Wink

**Affiliations:** 1 Department of Biology, Institute of Pharmacy and Molecular Biotechnology, Heidelberg University, Heidelberg, Germany; 2 School of Bio Sciences and Technology, VIT University, Vellore, India; German Cancer Research Center (DKFZ), GERMANY

## Abstract

Hairy root culture is a potential alternative to conventional mammalian cell culture to produce recombinant proteins due to its ease in protein recovery, low costs and absence of potentially human pathogenic contaminants. The current study focussed to develop a new platform of a hairy root culture system from *Nicotiana tabacum* for the production of recombinant human EPO (rhEPO), which is regularly produced in mammalian cells. The human *EPO* construct was amplified with C-terminal hexahistidine tag from a cDNA of Caco-2 cells. Two versions of rhEPO clones, with or without the N-terminal calreticulin (cal) fusion sequence, were produced by cloning the amplified construct into gateway binary vector pK_7_WG2D. Following *Agrobacterium rhizogenes* mediated transformation of tobacco explants; integration and expression of constructs in hairy roots were confirmed by several tests at DNA, RNA and protein levels. The amount of intracellular rhEPO from hairy root cultures with cal signal peptide was measured up to 66.75 ng g^-1^ of total soluble protein. The presence of the ER signal peptide (cal) was essential for the secretion of rhEPO into the spent medium; no protein was detected from hairy root cultures without ER signal peptide. The addition of polyvinylpyrrolidone enhanced the stabilization of secreted rhEPO leading to a 5.6 fold increase to a maximum concentration of 185.48 pg rhEPO^HR^ g^-1^ FW hairy root cultures. The rhizo-secreted rhEPO was separated by HPLC and its biological activity was confirmed by testing distinct parameters for proliferation and survival in retinal pigment epithelial cells (ARPE). In addition, the rhEPO was detected to an amount 14.8 ng g^-1^ of total soluble leaf protein in transgenic T_0_ generation plantlets regenerated from hairy root cultures with cal signal peptide.

## Introduction

Erythropoietin (EPO) is a glycoprotein hormone, which influences the production of erythrocytes through a process called erythropoiesis [1]. It is a common medicine for the treatment of anemia especially due to severe kidney failures [1]. It has a rapid response to all dialysis patients in the clinical trials and the patients became independent of blood transfusion [2]. It also exhibits parallel functions corresponding to erythropoiesis in non-erythropoietic tissues such as proliferation and differentiation [3]. It has additional protective functions in various cell types [4]. The major pleiotropic actions of EPO include neuroprotection in brain [5], cardiovascular protection [6] and retinal neuroprotection [7], which attracts the interest of scientific community. In the history of sports, EPO was used as a doping agent to improve the physiological performance in athletes of endurance sports [8]. Due to these applications, the research in synthesis and function of recombinant human erythropoietin (rhEPO) is extremely appreciated by pharmaceutical companies, research and academic institutions. Europe, North America and Asia-pacific are the major continents, which are widely engaged in the manufacture of rhEPO. Amgen Inc., Roche Diagnostics, Hospira Inc., are the key companies that actively participate in rhEPO market. RhEPO is formulated as gel, ointment, capsule or syrup and marketed by around 25 companies. The types of rhEPO that are highly promoted in the global pharmaceutical market are Epoietin (alfa, beta, gamma, omega, and delta) and darbopoietin-alfa.

Production of rhEPO was successfully achieved in prokaryotic cells (*Escherichia coli)* [9], eukaryotic cells such as *Pichia pastoris* [10] and the standard CHO mammalian cells [11]. The increasing demand for rhEPO in the pharmaceutical market are unlikely to be fulfilled with the limited potential and high cost of production from conventional mammalian cell cultures [12]. In order to overcome these limitations, plants as bioreactors may offer an alternative system for the production of pharmaceutically valuable rhEPO. The plant systems reported for the expression of rhEPO are cultured cells of tobacco [12]–[14], tobacco plants (*N*. *tabacum*) [15]–[18], *Nicotiana benthamiana* [19], *Medicago truncatula* [18], *Arabidopsis* [18], [20] and the moss *Physcomitrella patens* [21]. The production of recombinant therapeutic proteins is favoured in tobacco plants due to their fast growth, rapid reproduction and maintenance of genetic stability [22]. The tobacco plants are a naturally biosafe non-feed crop which can be easily used for the production of plant made therapeutics by minimizing the risk of contamination in the food chain [23].

The major advantage of plant expression systems is the high scale of consistent production of recombinant proteins and their ability to perform post-translational modification similar to mammalian systems [24]. Rhizosecretion, i.e. the secretion of proteins from roots, is an attractive new technology for the production of recombinant proteins from transgenic plants. Highly branched hairy roots, which are covered with tiny root hairs, represent an interesting plant tissue culture system for the continuous production of recombinant proteins and secondary metabolites [25]. These cultures can grow on Murashige Skoog (MS) media [26] with sugars and do not require any hormones or vitamins for their growth [27]. Root cultures can be maintained in bioreactors for a prolonged period [28]. The secretion of recombinant proteins into the culture medium of hairy root cultures attributes to the reduced cost of production due to the ease of downstream process and enhanced yield of protein recovery. The pharmaceutical proteins purified from hairy root cultures are less likely contaminated with human viral pathogens [29].

A few examples for the expression and secretion of recombinant proteins from tobacco hairy root cultures are xylanase of the bacterium *Clostridium thermocellum*, human placental alkaline phosphatase (SEAP), green fluorescent protein (GFP) of the jellyfish *Aequorea victoria* [30] and heat inducible β-glucuronidase [31]. The transformation of plants with *A*. *rhizogenes* has attracted interest because of the ease of plantlet regeneration from the plant tissues transformed with root inducing (Ri) T-DNA. Earlier reports in *Catharanthus roseus* [32] and *Solidago nemoralis* [33] are the few examples for the regeneration of plants from hairy root cultures produced through *A*. *rhizogenes* mediated transformation. The T-DNA integration of the expression plasmid into the plant genome facilitates normal differentiation of transformed callus or roots to regenerate into whole plants [34], [35]. This type of mechanism allows the transfer of genetic information encoded in the expression construct to the consecutive generations [36].

Herein, we report an improved hairy root culture system of tobacco (*Nicotiana tabacum*) for the expression and secretion of biologically active rhEPO. Leaf explants of tobacco were transformed with *A*. *rhizogenes* containing the *EPO* expression constructs with or without cal signal sequence. The cal signal sequence was fused to the *EPO* gene to facilitate the expression and secretion of rhEPO from the hairy root cultures (rhEPO^HR^). Biological activity of the rhEPO^HR^ secreted from hairy root cultures with cal signal peptide was determined using a retinal pigmented epithelial (ARPE) cells. Both integrity and *in vitro* biological efficacy of the rhEPO^HR^ secreted from hairy root cultures with cal signal peptide were shown to be similar to the commercially rhEPO, produced from mammalian CHO cells. Therefore, the present study report a simple hairy root culture system for the *in vitro* production of biologically active rhEPO^HR^.

## Materials and methods

### Cloning of *EPO*

Total RNA was isolated using TRIzol^®^ reagent (Invitrogen) from Caco-2 cells and the cDNA was produced using AMV reverse transcriptase (Promega). The *EPO* gene was amplified from cDNA using the gene specific primers F1 5′ GCCGAGCTTCCCGGGATG 3′ and R1 5′ GAGTGAGCTCAGGCGTCTTC 3′. PCR was performed with the initial denaturation at 94°C for 5 min, followed by 35 cycles of 94°C for 45 sec denaturation, 57°C for 45 sec annealing, 72°C for 1 min extension and with the final extension at 72°C for 7 min. The amplified product was purified using Nucleospin extract columns (Macheray-Nagel). The amplicon of 0.958 kb size was cloned in pDrive cloning vector (Qiagen) and the recombinant plasmid ^pDrive^rh*EPO* was introduced into *E*. *coli* strain DH5α through electroporation (Gene Pulser, Biorad). The recombinant plasmid ^pDrive^rh*EPO* was sequenced with M13 forward 5′ GTAAAACGACGGCCAG 3′ and reverse primer 5′ CAGGAAACAGCTATGAC 3′ by using the DYEnamic ET Terminator Cycle Sequencing Kit (GE Healthcare) and MegaBACE^™^ 1000 sequencer (GE Healthcare). The resulting sequences were compared with the nucleotide sequence of *EPO* transcript (ENSG00000130427) from the Ensembl database in pair-wise alignment using ClustalW software.

### Vector construction for expression of *EPO*

The Gateway^®^ cloning technology was developed by Invitrogen to facilitate rapid cloning of DNA fragments into multiple expression plasmids. The compatible binary vectors of the Gateway^®^ technology were acquired from VIB, Ghent (Belgium). In the present study, the binary plasmid (pK_7_WG2D) was used for the production of expression constructs with or without cal signal sequence. The plasmid ^pDrive^rh*EPO* was used as a template to amplify *EPO* cDNA for the production of expression clones.

The previously reported [37] binary expression plasmid incorporated with cal signal sequence (^pk7^cal) was employed in this study. The expression plasmid containing cal signal sequence (^pK7^cal/rh*EPO*) was generated by the ligation of amplified *EPO* cDNA in specific restriction sites of ^pk7^cal plasmid. The primers F2 designed with SacI restriction site 5’ GG*GAGCTC*ATGGGGGTGCACGAATGTCCTG 3’ and R2 designed with HindIII restriction site 5’ G*GAAGCTT*TCAGTGGTGGTGGTGGTGGTGTCTGTCC CCTGTCC 3′ were used to amplify the *EPO* gene. The restriction sites and six histidine tag sequences in the primers are shown in italicized and underlined characters respectively. The cal signal peptide was N-terminally fused to *EPO* gene in the expression construct ^pK7^cal/rh*EPO*.

The construct for *EPO* without the cal signal (^pK7^rh*EPO)* was generated by Gateway^™^ technology using primers F3 5’ *GGGGACAAGTTTGTACAAAAAAGCAGG CTTCGAAGGAGATAGAAC*ATGGGGGTGCACGAATGTCCTG 3’ and R3 5’ *GGGACCACTTTGTACAAGAAAGCTGGGTCTCA*GTGGTGGTGGTGGTGGTGTCTGTCCCCTG TCCTGCAG 3’. The sequences of “attB” site and six-histidine tag in the primers were depicted in italicized and underlined characters respectively. The “attB” sites were added according to the kit manufacturer’s instructions.

### Electrotransformation of expression constructs into *Agrobacterium*

The expression constructs with or without cal signal sequence was transferred into *A*. *rhizogenes* (*ATCC15834*) through electroporation using Gene Pulser (Bio-Rad). An aliquot of the reaction mixture was incubated overnight at 28°C in a selective Yeast Mannitol Broth (YMB) medium [38] with spectinomycin (50 mg L^-1^). Colony PCR was performed with primers (P35S 5’ ACAATCCCACTATCCTTC 3’ and T35S 5’ TCTGGG AACTACTCACAC 3’) to analyse the plasmid uptake. The positive clones were confirmed by sequencing (cloning of human *EPO* section) the plasmids using P35S and T35S primers (data not shown).

### *Agrobacterium rhizogenes*-mediated transformation of *N*. *tabacum*

The transformed *A*. *rhizogenes* were grown overnight at 28°C in YMB medium containing spectinomycin (50 mg L^-1^). The non-transformed *A*. *rhizogenes* (*ATCC 15834*) were grown in YMB medium containing no antibiotic and were used as a control. The cells were collected by centrifugation and were resuspended to OD_600_ = 1.0 using ½ MS [26] broth. Explants (discs of 0.5–1 cm^2^**)** were excised from mature leaves of *N*. *tabacum*. The explants were incubated with transformed or non-transformed *A*. *rhizogenes* at room temperature for 30 min in gentle shaking for co-infection. Then, the explants were incubated for 2–3 d in dark on solid Woody Plant Medium (WPM) [39].

After this period of incubation, the explants infected with transformed *A*. *rhizogenes* were transferred to solid WPM containing kanamycin (50 mg L^-1^) and cefotoxime (100 mg L^-1^) for the initiation of hairy root clones ^pK7^cal/rh*EPO*^HR^ or ^pK7^rh*EPO*^HR^ (with or without cal signal sequence respectively). The tobacco explants infected with non-transformed *A*. *rhizogenes* were transferred to solid WPM containing cefotoxime (100 mg L^-1^) for the initiation of control hairy root cultures Nt/ATCC^HR^. The explants were incubated in 25°C ± 2, photosynthetically active radiation (PAR) of 50 μm m^-2^ s^-1^ (Philips Master TL-D 30W/840) and 16 h photoperiod for the induction of hairy roots which occurred within 15 d. The hairy roots initiated (1–2 cm) were excised and propagated in 50 mL of liquid WPM in presence of respective antibiotics as explained above. The hairy root cultures were incubated in a platform shaker (New Brunswick^™^ Innova^®^ 2300) at 60 rpm and 25°C ± 2 under dark condition for further analysis. Growth kinetics of both the transformed and control hairy root cultures was determined for a maximum of 35 d. The hairy root clones (^pK7^cal/rh*EPO*^HR^ or ^pK7^rh*EPO*^HR^) were examined for kanamycin resistance, presence of *EPO* in plant genomic DNA, GFP fluorescence and expression of rhEPO^HR^ protein. The hairy root clones Nt/ATCC^HR^ served as a control in further experiments.

### Regeneration of T_0_ plantlets

Calli were induced from 15 d old hairy root cultures by placing them in callus induction (CI) medium [MS medium containing 2 mg L^-1^ 2,4-dichlorophenoxyacetic acid (2,4-D) and 1 mg L^-1^ kinetin] and were grown at 25°C ± 2 with the same light source as above in 16 h photoperiod. The calli were maintained by changing the CI medium every 2 week. After a period of 4 week, the transgenic calli were transferred to a regeneration medium containing MS medium with 2 mg L^-1^ 6-benzylaminopurine (BAP), 0.1 mg L^-1^ indole-3-acetic acid (IAA), 2% w/v sucrose and 50 mg L^-1^ kanamycin to produce regenerated transgenic T_0_ plantlets ^pK7^cal/rh*EPO*^RP^ or ^pK7^rh*EPO*^RP^. The control T_0_ plantlets Nt/ATCC^RP^ were regenerated by culturing the control calli in the regeneration medium with no antibiotic. The calli were regenerated into plantlets of 2–3 cm long plantlets within 4–6 week. Then, the regenerated T_0_ plantlets were transferred to a basal MS medium with kanamycin (50 mg L^-1^) for ^pK7^cal/rh*EPO*^RP^ or without kanamycin for Nt/ATCC^RP^ to attain normal growth. The plants were grown under same culture conditions in 16 h photoperiod for 4 week.

### Analysis of GFP expression in transformed plant tissues

The expression vector contains GFP which was used as a rapid marker to monitor the expression of rhEPO^HR^ from hairy root cultures (^pK7^cal/rh*EPO*^HR^ or ^pK7^rh*EPO*^HR^) and from leaves of regenerated transgenic plantlets (^pK7^cal/rh*EPO*^RP^ or ^pK7^rh*EPO*^RP^). The hairy root cultures Nt/ATCC^HR^ and the leaves of regenerated plantlets Nt/ATCC^RP^ served as a control. The sectioned samples of 10–20 d old hairy root cultures and 4 week old leaves from the regenerated plantlets were analysed for intracellular expression of GFP through confocal microscopy (Carl-Zeiss LSM 700).

### Genomic DNA analysis

#### Analysis of *EPO*, *rolC* and *virC* genes in hairy roots by PCR

The genomic DNA was isolated from 15–20 d old hairy root cultures and the PCR was performed with *virC*, *rolC* and *EPO* specific primers. F2 and R2 primers were used to amplify the *EPO* gene. The specific primers used for the amplification of *rolC* genes were *rolC*_F 5′ ATGGCTGAAGACGACCTGTGTT 3′ and *rolC*_R 5′ TTAGCCGATTGCA AACTTGCAC 3′. The specific primers used to amplify *virC* genes were *virC*_F 5′ ATCATTTGTAGCGACT 3′ and *virC*_R 5′ AGCTCAAACCTGCTTC 3′. Gel electrophoresis was performed for all the three PCR products from hairy root cultures (^pK7^cal/rh*EPO*^HR^ or ^pK7^rh*EPO*^HR^) and hairy root control culture (Nt/ATCC^HR^). The hairy root cultures confirmed the specifc signal for *rolC* and *EPO* specific primers were chosen for further analysis. The absence of specific signal for *virC* specific primers indicates the absence of bacterium (data not shown).

#### Southern blot analysis

**Fig 1:**
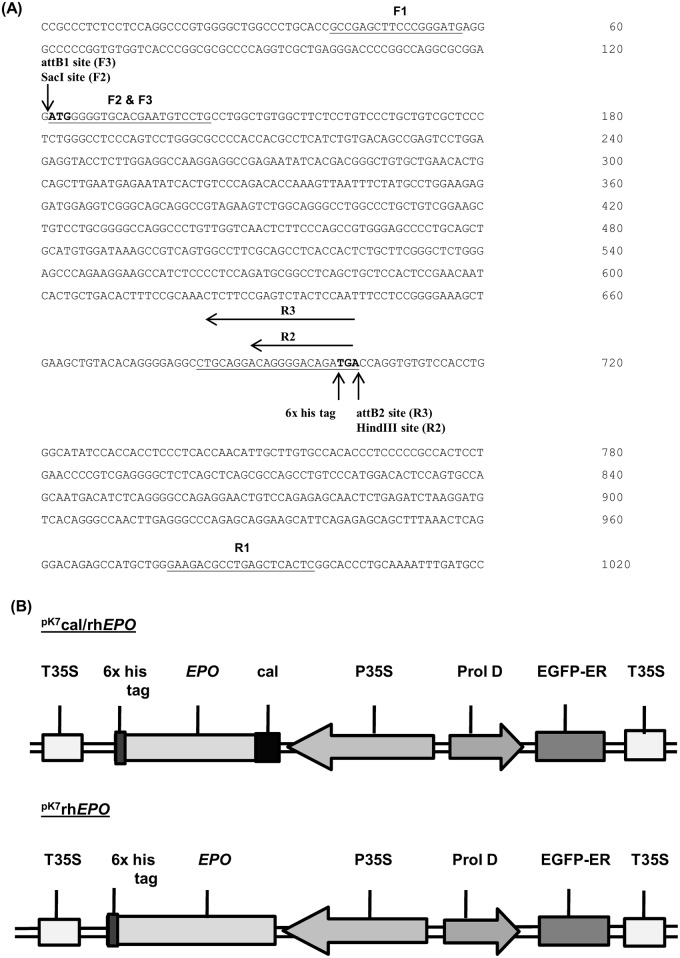
Cloning of human erythropoietin. (A) Illustration of different primer combinations used for amplification and cloning of *EPO* gene. The initiation (ATG) and termination (TGA) codons of translation are represented in bold characters. (B) Schematic representation of the T-DNA segments in *EPO* expression constructs with or without cal signal sequence (^pK7^cal/rh*EPO* or ^pK7^rh*EPO* respectively). The transcription of *EPO* gene is controlled by P35S promoter and T35S terminator. EGFP-ER is transcriptionally controlled by Prol D promoter and T35S terminator. The cal signal peptide is fused to the N-terminus of *EPO* in the ^pK7^cal/rh*EPO* construct.

Genomic DNA (10 μg) from hairy root cultures (^pK7^cal/rh*EPO*^HR^ or ^pK7^rh*EPO*^HR^) and control hairy root culture (Nt/ATCC^HR^) were digested with Hind III restriction endonuclease. The digested products were separated by electrophoresis in 0.8% w/v agarose. Then, the DNA in agarose gel was transferred to a Hybond-N^+^ nylon membrane (Amersham Biosciences) through capillary transfer for overnight at room temperature. The probe (0.6 kb) was produced by PCR using F2 and R2 primers (Fig 1A) and the conditions for PCR were explained above (cloning of human *EPO* section). In this analysis, DIG (Digoxigenin) labelled probe (PCR DIG Labelling kit, Roche) was used to study the integration of *EPO* in plant genomic DNA. Pre-hybridization of the membrane was performed at 60°C for 2 h using 20X SSC (sodium chloride and sodium citrate) buffer. Hybridization with *EPO* DIG labelled probe was performed at 60°C for 12 h. The membrane was washed with 2X SSC at room temperature. The hybridized probes were immunodetected with anti-DIG-AP (anti-DIG conjugated with alkaline phosphatase enzyme) using the substrate (CSPD) for chemiluminescent reaction. Then, the membrane was exposed to an X-ray film for 15–30 min.

### Transcript analysis of *EPO*

#### Reverse transcription PCR (RT-PCR)

RNA was isolated from 15 d old hairy root cultures ^pK7^cal/rh*EPO*^HR^ or ^pK7^rh*EPO*^HR^. The hairy root culture Nt/ATCC^HR^ was used as a negative control. Total RNA was isolated using TRIzol reagent (Invitrogen). The cDNA was produced using AMV reverse transcriptase (Promega) and ImProm-II^™^ Reverse Transcription System (Promega). The *EPO* cDNA was amplified using F2 and R2 gene specific primers (Fig 1A). The internal control *β actin* gene (GenBank X63603.1) was amplified using the primers NtAct forward 5’ GCTATTCAGGCTGTCCTTTCCTTGTATG 3’ and NtAct reverse 5’ CCGATATCAACATCACACTTCATAATG 3’. PCR conditions for the amplification of *EPO* and *β actin* genes were the same as explained above (cloning of human *EPO* section).

### Analysis of rhEPO^HR^ expression

#### Protein preparation from hairy root cultures

For the extraction of intracellular total protein, the fresh hairy root cultures (1 g) were collected and were powdered in presence of liquid nitrogen. Then it was homogenized using 1 ml of protein extraction buffer (300 mM sodium chloride, 50 mM sodium dihydrogen phosphate, 0.1% v/v Tween-20, 5 mM phenylmethyl sulfonyl fluoride, pH 8). The resulting lysate was sonicated (Bandelin Sonorex RK514) for 5 min. Then it was centrifuged to clarify the protein supernatant. The clarified proteins were used for Western blot and ELISA analysis. For the preparation of extracellular total protein, the spent medium of the hairy root clones were collected directly and were used for ELISA analysis. Then, the protein samples were affinity purified using Ni-TED columns (discussed later). The purified proteins were used for HPLC analysis and biological assays.

#### Western blot analysis for the expression of rhEPO^HR^

The intracellular and extracellular total protein from the 20 d old hairy root cultures ^pK7^cal/rh*EPO*^HR^or ^pK7^rh*EPO*^HR^ were separated by 12% w/v SDS-polyacrylamide gel electrophoresis (SDS-PAGE). The total soluble intracellular protein from the hairy root culture Nt/ATCC^HR^ was employed as a negative control. Using a semi-dry blotting apparatus (Fischer), the proteins were then transferred to a polyvinylidene fluoride (PVDF) membrane (Millipore). After the transfer of proteins, the membrane was blocked with 5% w/v non-fat dry milk in PBS-T (phosphate buffered saline with 0.1% v/v Tween 20) for 1h at 4°C. The membrane was then incubated with 1:5000 diluted rabbit erythropoietin antiserum (E2531, Sigma-Aldrich) and 1:10000 diluted goat antirabbit horseradish peroxidase (HRP) conjugate (A0545, Sigma-Aldrich) for immunological reactions. Western Lightning Chemiluminscence Reagent (Perkin Elmer) was prepared and the membrane was developed using an X-ray developer (Fujifilm).

#### Quantification of rhEPO^HR^ by ELISA

The concentration of rhEPO^HR^ in cell lysates and spent medium of hairy root culture ^pK7^cal/rh*EPO*^HR^ with cal signal sequence was determined using an ELISA kit (Ray Biotech). The intracellular concentration of rhEPO^HR^ in 4 week old leaves of regenerated T_0_ plantlet ^pK7^cal/rh*EPO*^RP^ was also detected in this assay. Standards ranging from 0.1 to 100 mIU rhEPO mL^-1^ (1 IU = 10 ng) were included in the kit. The standards and the test samples (total protein samples from hairy root cultures and leaves of regenerated plantlets) were analysed in wells pre-coated with EPO antibody. A volume of 100 μL from a 40 μg mL^-1^ stock solution was added to each well in order to obtain absorbance values in detectable range of concentrations for the standard EPO provided by the kit manufacturer. The samples were incubated for 2.5 h in the pre-coated wells. The wells were washed by using wash buffer (provided in the kit) to remove unbound protein in the wells. In the next step, biotinylated human erythropoietin antibody was added and was incubated for 1 h. Again, the wells were washed to remove the unbound biotinylated antibody. It is followed by the addition of HRP-streptavidin conjugate to the wells and the wells were incubated for 45 min. The wells were washed again and substrate 3,3’,5,5’- tetra methyl benzidine (TMB) was added. The wells were incubated for colour development. In the next step, the stop solution (provided in the kit) was added to the wells and the absorbance was measured at 450 nm. The concentration of rhEPO^HR^ in the samples was determined using GraphPad Prism 5 by comparing the values of absorbance.

#### Affinity chromatography for the purification of extracellular rhEPO^HR^

The spent medium from hairy root culture ^pK7^cal/rh*EPO*^HR^ or control hairy root culture Nt/ATCC^HR^ was precipitated with 60% w/v of ammonium sulphate. The precipitated protein was resuspended with LEW buffer (300 mM sodium chloride, 50 mM sodium phosphate, pH 8) which was followed by desalting through Sephadex 25 columns (Genei). Finally, it was purified using Ni-TED columns (polyhistidine protein purification kit, Genei). The purified proteins were used for HPLC analysis and biological assays.

#### HPLC analysis of rhEPO^HR^

A volume of 20 μL (from 0.1 μg mL^-1^) from the affinity purified proteins of the hairy root cultures ^pK7^cal/rh*EPO*^HR^ and the control hairy root cultures Nt/ATCC^HR^ were analysed by HPLC. The analysis was performed in YoungLin HPLC system (ACME 9000) using a Kromasil 100-5C18 column (250 x 4.6 mm length), SP930D gradient pump and UV730D detector. The isocratic separation was carried out with 150 mM phosphate buffer, pH 7.0 with a flow rate of 0.35 mL/min. The parameters were selected according to the instructions of Agilent Technologies for separating rhEPO expressed in CHO cell lines (rhEPO^CHO^). The peak for 10 ng of standard EPO (Sigma-Aldrich) was recorded at 225 nm wavelength and the peak area of the test samples were compared accordingly.

### Bioactivity of recombinant rhEPO^HR^

#### Preparation of a retinal pigmented epithelial (ARPE) cells

ARPE cells (10^3^ cells/well) were grown in DMEM supplemented with 10% v/v FCS (Sigma-Aldrich). After the cells had reached 80–90% confluence, they were grown in serum-reduced medium (DMEM supplemented with 2% v/v FCS) for 24 h. It was followed by the addition of different concentrations (serial dilutions ranging from 10 ng mL^-1^ to 0.001 ng mL^-1^) of rhEPO^HR^ to the cells and the cells were grown for 24 h at 37°C in CO_2_ incubator. All the samples in these assays were performed in triplicates.

#### MTT assay

EPO activity on cell viability was measured using MTT (3-(4,5-dimethylthiazol-2-yl)-2,5-diphenyl tetrazolium bromide), (Sigma-Aldrich). The treated cells (as explained above) were washed with 1x PBS. 100 μL of MTT (5 mg mL^-1^ PBS) was added to each well and the cells were kept overnight at 37°C in CO_2_ incubator. After this incubation, 100 μL of DMSO was added to dissolve the crystals and the absorbance was measured at 570 nm using a microplate reader (Bio-Rad). The values of absorbance were plotted against different concentrations of rhEPO^HR^.

#### BrdU proliferation assay

Bromodeoxyuridine (BrdU) is an analogue to thymidine that binds to DNA of the newly proliferating cells. The BrdU proliferation assay was performed according to the instructions of manufacturer (CytoSelect^™^ BrdU Cell Proliferation ELISA Kit, Cell Bio labs Inc.). The cells treated with rhEPO^HR^ were prepared as discussed above in MTT assay. 10 μL of BrdU labelling solution (diluted with growth medium) in a ratio of 1:100 was added to the treated cells in each well. Cells were kept at 37°C for 2 hours in CO_2_ incubator. After this step, 200 μL of FixDenat solution was added to each well and the cells were incubated for 30 min at 30°C. Both the BrdU labelling solution and FixDenat solution were removed. A volume of 100 μL anti-BrdU-POD (POD- peroxidise) was added to each well and was incubated at room temperature for 90 min. After washing with wash buffer (provided in kit), 100 μL of substrate solution (provided in kit) was added to each well. The reactions were incubated at room temperature for 30 min until colour development. The absorbance was recorded without stop solution at 405 nm and was recorded with stop solution at 450 nm. The difference in the two absorbance values was considered for further calculations and was used for plotting graph.

## Results and discussion

### Cloning of human *EPO*

In order to express biologically active rhEPO^HR^ in *N*. *tabacum*, the *EPO* cDNA of 0.958 kb was amplified from Caco-2 cells using F1 and R1 primers (Fig 1A). The amplified product was cloned in a pDrive (Qiagen) cloning vector, which was used as a template for sub-cloning in plant binary expression vector pK_7_WG2D. The gene specific F2 and R2 primers were used for the production of *EPO* expression construct ^pK7^cal/rh*EPO* with cal signal sequence (Fig 1A). The gene specific F3 and R3 primers were used to produce *EPO* expression construct ^pK7^rh*EPO* without cal signal sequence (Fig 1A). The expression constructs with or without cal signal sequence were constructed using Gateway technology^™^ (Fig 1). This technology involves recombination events developed from Phage Lambda Integrase [40] and is commercially available as Gateway recombination cloning technology by Invitrogen. It provides an advanced, effective and reliable cloning technology as compared to the conventional cloning methodology [41]. An illustration of the T-DNA of the expression constructs with or without (^pK7^cal/rh*EPO* or ^pK7^rh*EPO* respectively) is shown in Fig 1B. The fusion of cal signal with *EPO* directs the expressed protein to the secretory pathway of plant. The histidine tag fused to *EPO* facilitates single-step purification of the recombinant protein. The integration of T-DNA constructs into the plant genome was monitored by GFP expression.

Previously, the signal peptide of native human *EPO* was incorporated in the expression constructs for the secretion of rhEPO in tobacco plant [12] and cultured BY2 cells of tobacco [13]. In this study, the cal signal sequence was fused to the N-terminus of *EPO* gene sequence in the expression construct ^pK7^cal/rh*EPO*^HR^ (Fig 1B) to direct expressed recombinant protein to the plant secretory pathway. The cal signal peptide incurred the cytoplasmic stability and ER transport of rhEPO^HR^ expressed from the hairy root culture ^pK7^cal/rh*EPO*^HR^. The disulphide bridges are formed in ER that is essential for proper protein folding [42]. In this study, the transcription of *EPO* gene controlled by CaMV 35S promoter produced substantial amount of rhEPO^HR^ from hairy root cultures. According to previous reports, the constitutive promoter CaMV 35S confers high level of expression of recombinant proteins by regulating the mechanism of transcription [43].

### *Agrobacterium* mediated transformation and growth kinetics of the hairy root cultures

**Fig 2:**
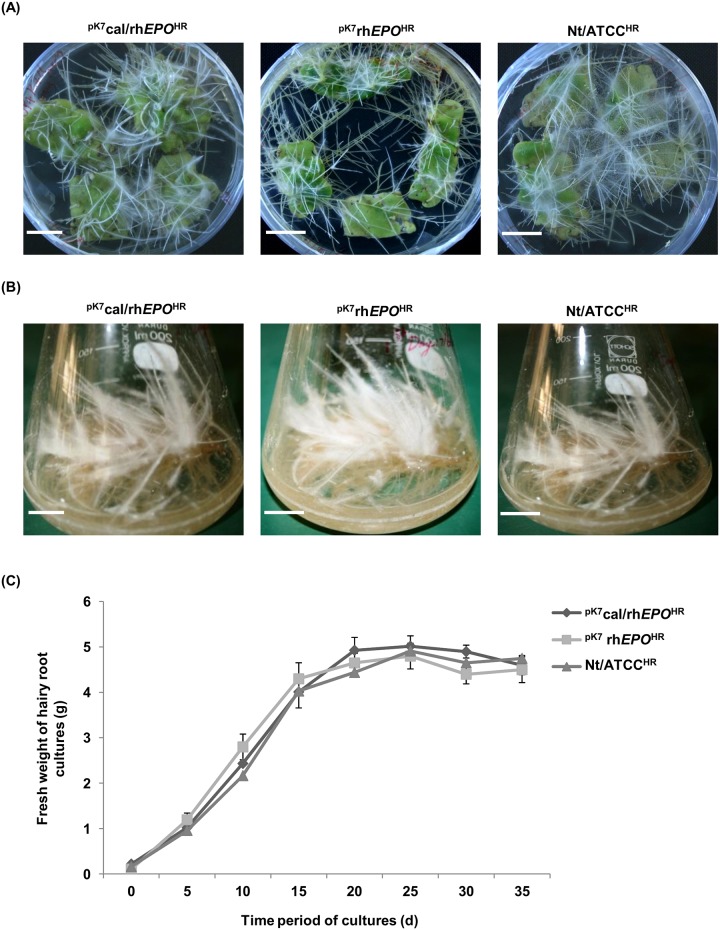
*Agrobacterium rhizogenes* mediated transformation and growth kinetics of the hairy root cultures. (A) Hairy root culture with cal signal sequence ^pK7^cal/rh*EPO*^HR^, hairy root culture without cal signal sequence ^pK7^rh*EPO*^HR^ and control hairy root cultures Nt/ATCC^HR^ were shown. Scale bars are 1 cm. (B) Establishment of the initiated hairy roots (1–2 cm) in 50 mL of liquid WPM. Scale bars are 5 cm. (C) Graphical representation of growth kinetics for the hairy root cultures. All samples were measured in triplicates and each data represents the average ± SD.

In next steps, the expression constructs (Fig 1B) with or without cal signal sequence were transformed into *A*. *rhizogenes*, which were used for the initiation of hairy root cultures ^pK7^cal/rh*EPO*^HR^ or ^pK7^rh*EPO*^HR^ (Fig 2A). The non-transformed *A*. *rhizogenes* cells were used for the production of control hairy root cultures Nt/ATCC^HR^. The leaf explants infected with transformed *A*. *rhizogenes* were placed in solid WPM containing kanamycin (50 mg L^-1^) and cefotoxime (100 mg L^-1^) for the selection of hairy root cultures ^pK7^cal/rh*EPO*^HR^ or ^pK7^rh*EPO*^HR^. The leaf explants infected with non-transformed *A*. *rhizogenes* were placed in solid WPM containing cefotoxime (100 mg L^-1^) for the selection of co ntrol hairy root cultures Nt/ATCC^HR^.

The growth kinetics of hairy root cultures as shown in Fig 2B was determined by measuring their fresh weights for a period of 35 d (Fig 2C). No significant difference in growth rate was observed between control and hairy root cultures. The cultures reached stationary growth after 25 d. After 30 d, the cultures turned brownish and gradually died within next 20 d.

### Regeneration of T_0_ plantlets

**Fig 3:**
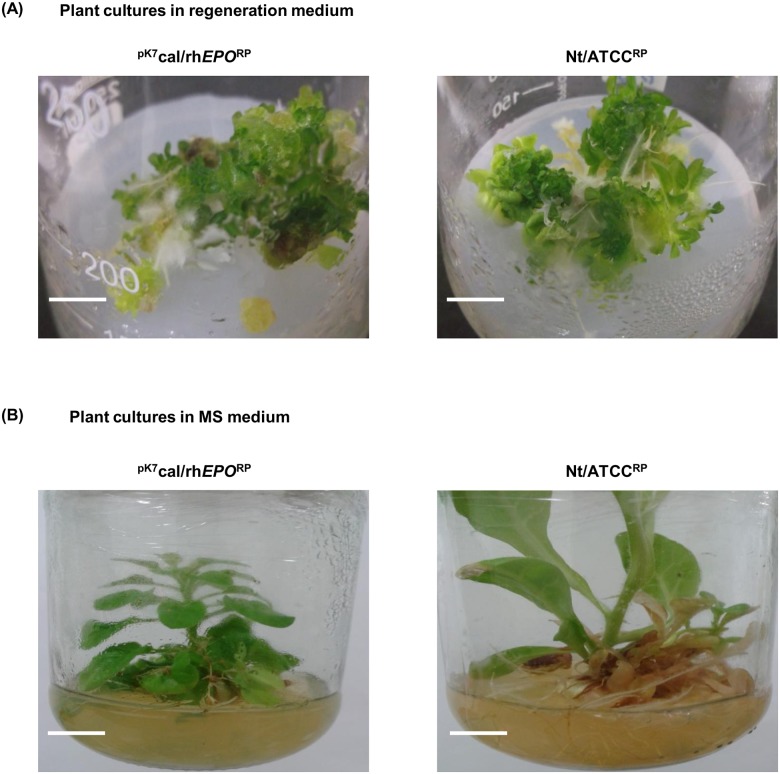
Regeneration of T_0_ tobacco plantlets. (A) The 2 week old transgenic or control calli were cultured in regeneration medium. Scale bars are 5.8 cm. (B) The 4 week old transgenic or control regenerated T_0_ plantlets (^pK7^cal/rh*EPO*^RP^ or Nt/ATCC^RP^ respectively) were grown in MS medium. Scale bars are 5.9 cm.

The transgenic calli initiated from 15 d old hairy root culture ^pK7^cal/rh*EPO*^HR^ were transferred to regeneration medium containing 50 mg L^-1^ kanamycin (Fig 3A). As a control, the calli were initiated from hairy root culture Nt/ATCC^HR^ and were transferred to the regeneration medium without antibiotic (Fig 3A). The concentration of plant hormones reported as in previous studies of Rahman et al. [44] was modified to 2 mg L^-1^ BAP and 1 mg L^-1^ IAA. The specified concentration of BAP and IAA were added along with 2 mg L^-1^ kinetin to the MS basal medium for the preparation of regeneration medium. The regenerated T_0_ transgenic plantlet ^pK7^cal/rh*EPO*^RP^ was transferred to MS medium with kanamycin and the control regenerated T_0_ plantlet Nt/ATCC^RP^ was transferred to MS medium with no antibiotic (Fig 3B). This protocol resulted in substantial initiation of callus and shoot regeneration from the hairy root cultures (Fig 3A and 3B).

The potency to regenerate whole plants from the hairy roots determines the completion of genetic transformation process [44]. In agreement with this, we have produced regenerated T_0_ plantlets ^pK7^cal/rh*EPO*^RP^ from hairy root cultures ^pK7^cal/rh*EPO*^HR^ (Fig 3A and 3B). The morphology of regenerated T_0_ plantlets had shown variations in growth of stems and leaves when compared to non-transformed plantlets (Fig 3B), which is similar to the reports of Cheon et al. [20].

### GFP expression in hairy root cultures and leaves of regenerated plants

**Fig 4:**
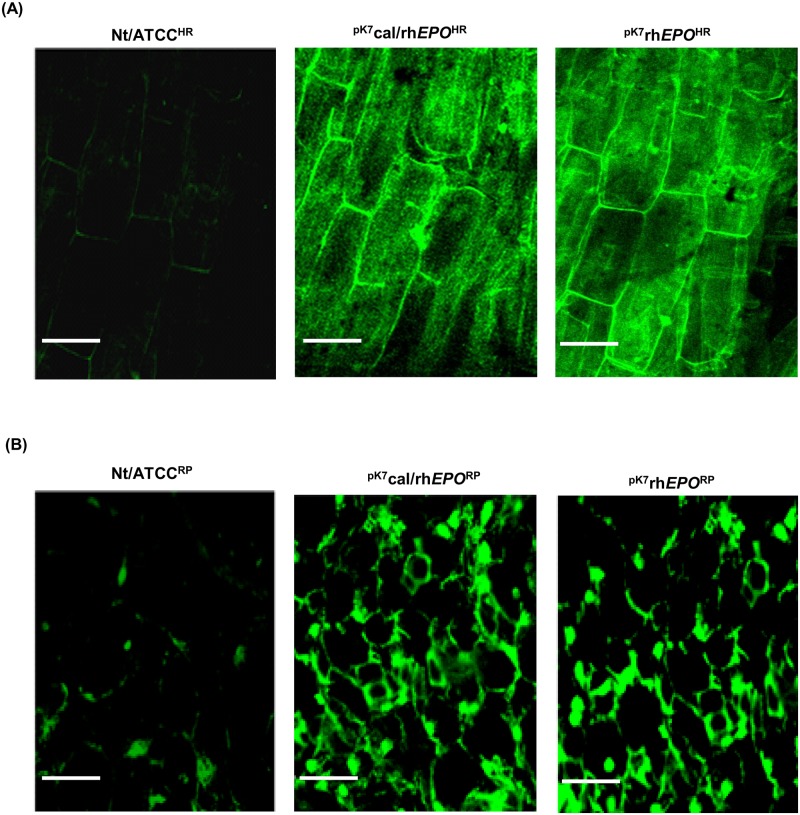
GFP expression in hairy root cultures and in leaves of regenerated plant. (A) Confocal images obtained from 15 d old hairy root cultures. (B) Confocal images obtained from 4 week old leaf tissues of regenerated T_0_ transgenic plantlets. Images are representative for three experiments and the scale bars are 50 μm.

In the first step of expression analysis, GFP fluorescence was observed from hairy root cultures ^pK7^cal/rh*EPO*^HR^ or ^pK7^rh*EPO*^HR^ (Fig 4A) and leaves of T_0_ plantlets ^pK7^cal/rh*EPO*^RP^ or ^pK7^rh*EPO*^RP^ (Fig 4B). Fluorescence analysis revealed that the hairy roots exhibited GFP expression as compared to the control hairy root culture Nt/ATCC^HR^, suggesting the successful integration of *EPO* expression constructs into tobacco genome. The leaves of regenerated T_0_ plantlets ^pK7^cal/rh*EPO*^RP^ or ^pK7^rh*EPO*^RP^ were also observed with GFP fluorescence as compared to the leaves from control regenerated T_0_ plants Nt/ATCC^RP^ (Fig 4B).

### Genomic DNA and transcript analysis of hairy root cultures

**Fig 5:**
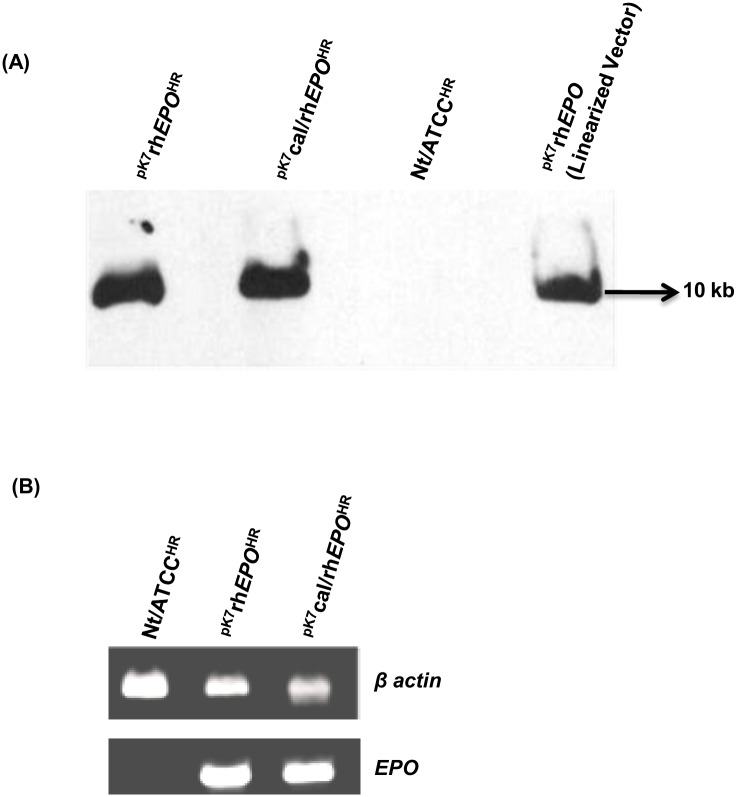
Genomic DNA and transcript analysis of hairy root cultures. (A) Southern blot analysis was performed using the genomic DNA of transformed hairy root clones. (B) RT-PCR analysis of *EPO* mRNA in hairy root cultures ^pK7^cal/rh*EPO*^HR^ and ^pK7^rh*EPO*^HR^. The presence of mRNA for tobacco *β actin* gene is shown as an internal control for transcript analysis.

In the next steps, the hairy root cultures were investigated for genomic integration and transcription of the anticipated *EPO* gene via Southern blot and RT-PCR respectively. A single copy integration of T-DNA into tobacco genomic DNA was detected in Southern blot analysis for hairy root cultures ^pK7^cal/rh*EPO*^HR^ and ^pK7^rh*EPO*^HR^ using DIG labelled *EPO* probes (Fig 5A). According to the reports of [45], [46], a single copy integration resulted in high and stable expression of a transgene (EPO). The specific signal was detected at an expected size of about 10 kb for the *EPO* expression plasmid without cal signal sequence (Fig 1B) and was used as a positive control. No signal was detected in the negative control hairy root culture Nt/ATCC^HR^.

**Fig 6:**
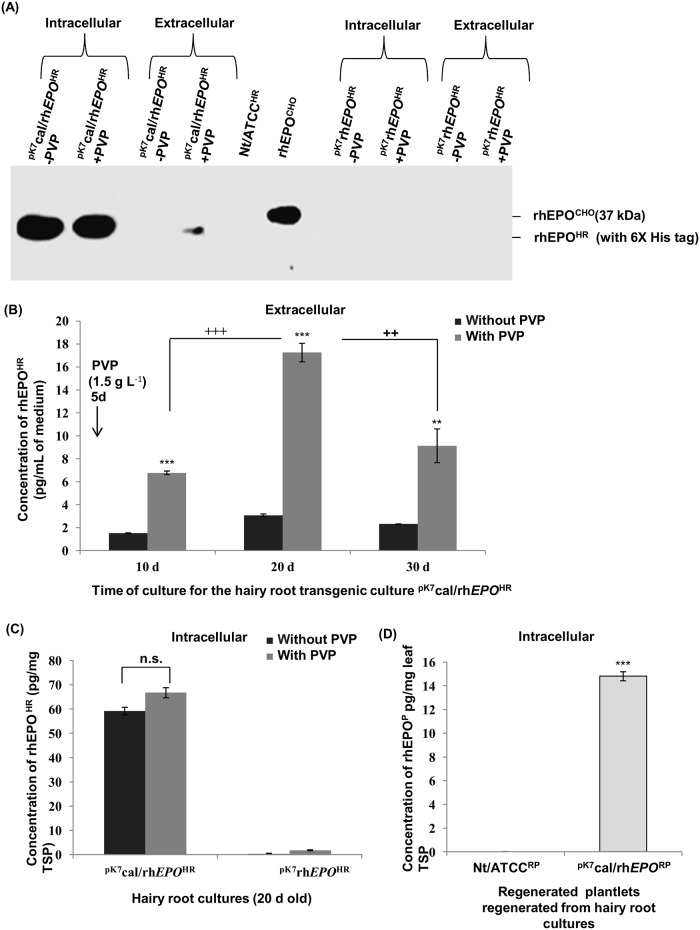
Western blot and ELISA analysis for effect of PVP treatment on the expression or yield of rhEPO. (A) Western blot analysis of intracellular and extracellular total protein was performed for 20 d old hairy root cultures following the addition of PVP to the growth medium. For size comparison, recombinant human erythropoietin rhEPO^CHO^ of molecular size 37 kDa from CHO cells (Sigma) was used. (B) ELISA analysis of extracellular rhEPO^HR^ concentration in hairy root culture ^pK7^cal/rh*EPO*^HR^ is shown as ng ml^-1^ for three different intervals 10, 20 and 35 d. (C) ELISA analysis of intracellular rhEPO^HR^ concentration for 20 d old hairy root culture ^pK7^cal/rh*EPO*^HR^ is shown as ng/μg of total soluble protein is shown as ng μg^-1^ of total soluble protein. (D) ELISA analysis of intracellular rhEPO^RP^ concentration in 4 w old leaf tissues of transgenic plants ^pK7^cal/rh*EPO*^RP^ regenerated from hairy root cultures ^pK7^cal/rh*EPO*^HR^ is shown as ng μg^-1^ of total soluble protein. Each bar represents the average ± SD in panels B–D. Student’s t-test was performed to analyze significance, N = 3, *** and ^+++^ p<0.001, ** and ^++^ p<0.01, and n.s. indicates no significance in panels B–D.

In the following steps, RT-PCR analysis was performed for the transformed clones. As shown in Fig 5B, *EPO* transcript of expected molecular size was observed in hairy root cultures ^pK7^cal/rh*EPO*^HR^ or ^pK7^rh*EPO*^HR^ but not in control hairy root culture Nt/ATCC^HR^. The product of transcript for the *β actin* gene was used as an internal control. The presence of transcripts provided the evidence for a successful transcription of integrated *EPO* gene in tobacco hairy roots. The rhEPO^HR^ protein was detected only in ^pK7^cal/rh*EPO*^HR^ with cal signal sequence (Fig 6A) regardless of the presence of *EPO* mRNA in both hairy root clones ^pK7^cal/rh*EPO*^HR^ and ^pK7^rh*EPO*^HR^ (Fig 5B). Similarly, the level of mRNA was detected without any significant difference in presence or absence of signal sequence when stable antibody expression was detected only in the presence of signal sequence in tobacco plants [47].

### Western blot and ELISA analysis for the effect of PVP treatment on expression or yield of rhEPO

Consequently, Western blot was performed using rabbit anti-human EPO (Sigma-Aldrich) to analyse intracellular and extracellular expression of rhEPO^HR^ in total protein samples from 20 d old hairy root cultures ^pK7^cal/rh*EPO*^HR^ or ^pK7^rh*EPO*^HR^ following the addition of PVP to the growth medium (Fig 6A). The hairy root culture Nt/ATCC^HR^ served as a negative control (Fig 6A). The expression of intracellular and extracellular rhEPO^HR^ was detected in hairy root cultures with cal signal ^pK7^cal/rh*EPO*^HR^. The molecular weight of rhEPO^HR^ expressed from hairy root cultures was lower than that of standard recombinant human erythropoietin from CHO cells (rhEPO^CHO^) presumably due to different pattern of sugar chains attached to the functional rhEPO^HR^ in plant expression system. The molecular size of rhEPO^HR^ protein expressed in hairy root culture ^pK7^cal/rh*EPO*^HR^ was detected around 33 kDa (Fig 6A) which resembled the size of previously reported rhEPO from plants or cultured cell lines of tobacco, *Medicago truncatula* and *Arabidopsis thaliana* [12]–[20].

The results of ELISA (Fig 6B and 6C) from intracellular and extracellular rhEPO^HR^ confirmed the results obtained in Western blot (Fig 6A) for hairy root cultures ^pK7^cal/rh*EPO*^HR^ or ^pK7^rh*EPO*^HR^. The concentration of intracellular and extracellular rhEPO^HR^ was determined in presence or absence of PVP in culture medium (Fig 6B and 6C). The concentration of intracellular rhEPO^HR^ analysis was examined for 20 d old hairy root culture ^pK7^cal/rh*EPO*^HR^ (Fig 6C) due to the detection of maximum amount of extracellular rhEPO^HR^ secretion as shown in Fig 6B. Hairy root cultures lacking cal signal sequence ^pK7^rh*EPO*^HR^ served as a control. As expected, the rhEPO^HR^ protein was not detected in cell extracts and spent medium of hairy root culture without cal signal ^pK7^rh*EPO*^HR^. Similarly, the rhEPO^HR^ was not observed in the cell extracts of negative controls (hairy root culture Nt/ATCC^HR^). In case of ^pK7^rh*EPO*^HR^ hairy root culture lacking cal signal sequence, intracellular rhEPO^HR^ protein was detected in ELISA (Fig 6C) but not in Western blot (Fig 6A). This result can be explained by the previous report that partially degraded protein or peptide fragments could be detected by ELISA quantitative analysis [48]. The low level of secretion of extracellular rhEPO^HR^ from hairy root culture ^pK7^cal/rh*EPO*^HR^ containing cal signal peptide may be attributed to the accumulation of recombinant protein inside apoplast. According to an earlier report, fusion of cal signal peptide to GFP resulted in higher level of GFP accumulation in apoplast than the level of GFP detected in hydroponic medium and cell extract [30]. The concentration of rhEPO^HR^ in total soluble protein (TSP) was higher than the concentration of secreted rhEPO^HR^ in spent medium of hairy root culture ^pK7^cal/rh*EPO*^HR^ (Fig 6B and 6C) apparently due to the retention of protein with molecular weight more than 20–30 kDa (rhEPO^HR^≈33 kDa) inside the apoplast of hairy roots [49].

The stability of rhEPO^RP^ expression was examined in regenerated plantlet ^pK7^cal/rh*EPO*^RP^ (Fig 6D). The plant Nt/ATCC^RP^ regenerated from the hairy root culture Nt/ATCC^HR^ served as a negative control (Fig 6D). According to ELISA analysis, the amount of intracellular human recombinant erythropoietin from the leaves of regenerated plantlets (rhEPO^RP^) was 14.8 pg rhEPO^RP^ mg^-1^ of total soluble leaf protein (Fig 6D). The significance of this study is a stable integration of *EPO* gene into the plant genome which would enable the regenerated plantlets to act as bioreactors for the production of biologically active rhEPO.

### HPLC analysis of rhEPO^HR^ in transformed hairy root cultures

**Fig 7:**
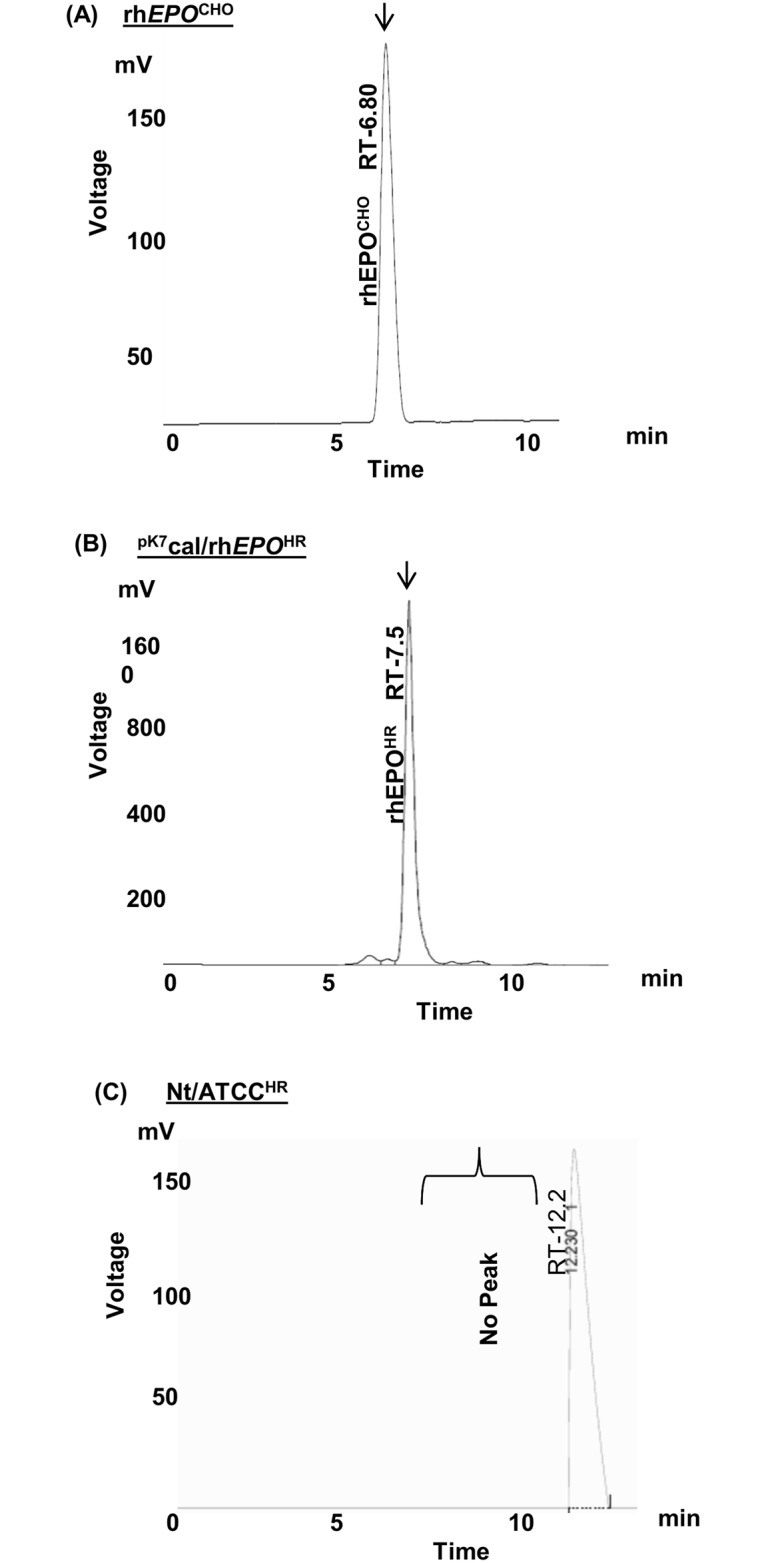
HPLC profile of rhEPO in transformed and control hairy root cultures. (A) Positive control rhEPO^CHO^ standard recombinant human erythropoietin from CHO cells. (B) Hairy root culture ^pK7^cal/rh*EPO*^HR^. (C) Negative control hairy root culture Nt/ATCC^HR^. The arrow indicates the presence of peaks.

HPLC analysis was employed to examine the chromatographic profile of secreted rhEPO^HR^ that was affinity purified from the spent medium of hairy root culture ^pK7^cal/rh*EPO*^HR^ with a cal signal sequence. The peak for standard EPO was obtained at 225 nm wavelength. For the analysis, the spent medium from transformed and control hairy root cultures was precipitated with 60% w/v ammonium sulphate and desalted using Sephadex G-25 columns. The precipitated proteins were purified using Ni-TED columns by affinity chromatography. In the hairy root culture a peak was recorded for ^pK7^cal/rh*EPO*^HR^ (Fig 7B) at 7.5 min, as compared to the retention time (Rt) at 6.8 min for positive control standard rhEPO^CHO^ (Fig 7A). The negative control hairy root cultures Nt/ATCC^HR^ showed no peak at the specified Rt. Furthermore, an additional peak was detected at RT 12.2 in both transformed ^pK7^cal/rh*EPO*^HR^ and control hairy root cultures Nt/ATCC^HR^ (Fig 7B and 7C), most likely due to impurities associated with Ni-TED affinity purification process.

### Bio-assay of rhEPO^HR^ in a retinal pigmented epithelial (ARPE) cells

After confirming and standardizing the conditions required for the secretion of rhEPO^HR^ in both hairy root cultures and in regenerated transgenic plantlets, we asked the question whether the secreted recombinant protein is functional in biological context. For this purpose, we studied the growth stimulation of ARPE cells by rhEPO^HR^. The cells were treated for 24 h in low serum (2% v/v FCS) containing DMEM basal medium with varying concentrations of affinity purified extracellular rhEPO^HR^ (ranging from pictogram to microgram per millilitre). The rhEPO^HR^ was purified through affinity chromatography using Ni-TED columns from the spent medium of hairy root culture ^pK7^cal/rh*EPO*^HR^ with cal signal sequence. As a control experiment, the cells were treated with standard recombinant human EPO from CHO cells (rhEPO^CHO^). Then, the treated cells were kept at 37°C in a CO_2_ incubator for 24 h. At the end of 24 h incubation, the cell viability or proliferation was measured by MTT assay or BrdU cell proliferation assay, respectively.

**Fig 8:**
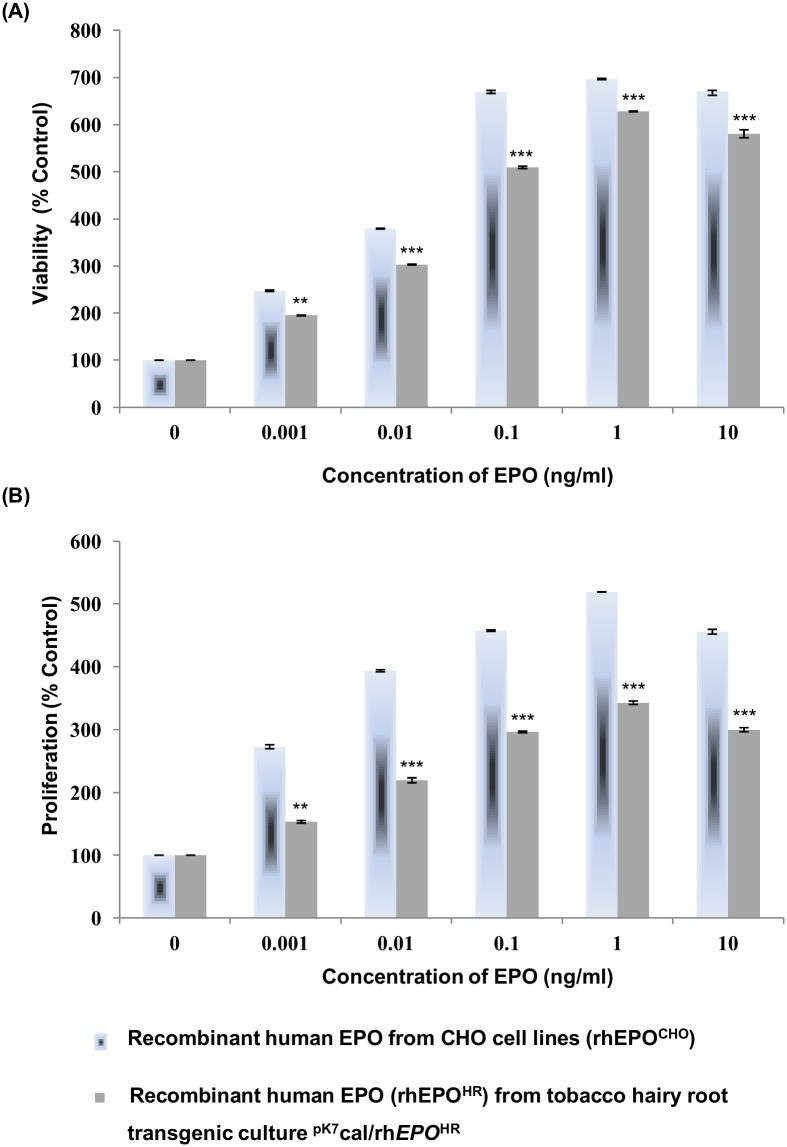
Bio-assay of rhEPO in a retinal pigmented epithelial (ARPE) cells. (A) Impact of rhEPO^HR^ on viability in ARPE cell lines was measured with MTT assay. (B) Impact of rhEPO^HR^ on proliferation of ARPE cells was analysed with BrdU cell proliferation assay. The results are presented as percentage of cell viability in Panel A and percentage of cell proliferation in panel B. Student’s t-test was performed for Panels A and B to analyze significance, N = 3, *** p<0.001 and ** p<0.01 as compared to the corresponding data of untreated group (grey bars).

As shown for MTT assay (Fig 8A), rhEPO^HR^ secreted from the hairy root culture ^pK7^cal/rh*EPO*^HR^ promoted dose-dependent growth of ARPE cells. A concentration of 1 ng rhEPO^HR^ mL^-1^ induced a maximal growth of 6.4 fold as compared to the cells cultured in the absence of rhEPO^HR^. To complement the observations, we performed a proliferation assay by employing BrdU (Fig 8B). Analysis of colorimetric signals as a marker for cell proliferation revealed that cells treated with secreted rhEPO^HR^ from ^pK7^cal/rh*EPO*^HR^ showed dose-dependent cell proliferation to a maximum of 3.3 fold at a concentration of 1 ng rhEPO^HR^ mL^-1^ (Fig 8B). The comparative data between the cells treated with rhEPO^HR^ and standard rhEPO^CHO^ revealed that there was no significant difference in the magnitude of cell viability and proliferation (Fig 8). The *in vitro* biological activity was reported for rhEPO expressed from tobacco cultured cells [12], [13] and tobacco plants [50]. It is noteworthy that the rhEPO^HR^ secreted from hairy root cultures ^pK7^cal/rh*EPO*^HR^ with cal signal sequence was identified as functionally active *in vitro* with ARPE cells (Fig 8).

### Effect of PVP on the yield of extracellular rhEPO^HR^ protein from hairy root cultures of *N*. *tabacum*

Our result confirmed that the addition of PVP into culture medium increased the amount of extracellular rhEPO^HR^ from the hairy root culture ^pK7^cal/rh*EPO*^HR^, without revealing any significant effect in the concentration of intracellular rhEPO^HR^ (Fig 6B and 6C). The protocol for PVP treatment was adapted from a previous study [37]. The addition of 1.5 g L^-1^ PVP into the growth medium on day 5 of culture enhanced the amount of extracellular rhEPO^HR^ up to 17.25 pg rhEPO^HR^ mL^-1^ of spent medium from 20 d old hairy root cultures (^pK7^cal/rh*EPO*^HR^) (Fig 6B). Hence, the production level of extracellular rhEPO^HR^ from the spent medium of ^pK7^cal/rh*EPO*^HR^ achieved a 5.6 fold increase in presence of PVP as compared to the growth medium without PVP (Fig 6B). The amount of extracellular rhEPO^HR^ from the hairy root culture ^pK7^cal/rh*EPO*^HR^ reached a maximum during the stationary phase after 20 d of culture (Fig 6B). Then, it was gradually declined after 25 d of culture probably due to factors such as protease activity in growth medium [48], [51] and low stability of protein in growth medium with minimum nutrients [52]. PVP did not affect the amount of intracellular rhEPO^HR^ from hairy root cultures ^pK7^cal/rh*EPO*^HR^ (Fig 6C). The concentration of intracellular rhEPO^HR^ was measured up to 66.75 pg rhEPO^HR^ mg^-1^ of TSP from hairy root culture ^pK7^cal/rh*EPO*^HR^ (Fig 6C).

### Production of rhEPO^HR^ in hairy root cultures of *N*. *tabacum*

The finding in the present study is the first report on expression and secretion of rhEPO^HR^ from hairy root cultures of *N*. *tabacum*, for which two different hairy root cultures of *N*. *tabacum* expressing human erythropoietin (rhEPO^HR^) with or without cal signal sequence (^pK7^cal/rhEPO^HR^ or ^pK7^rhEPO^HR^ respectively) through *A*. *rhizogenes* mediated plant transformation were established (Fig 2A). The intracellular rhEPO^HR^ protein was expressed with a production level up to 0.0066% (66.75 pg mg^-1^) of TSP from hairy root cultures with cal signal peptide ^pK7^cal/rh*EPO*^HR^ (Fig 6C). It is higher than the production of rhEPO reported as 0.0026% (26 pg mg^-1^) of TSP from the cultured BY2 cells of tobacco [14]. The secretion of extracellular rh*EPO*^HR^ was recorded to a maximum 185.48 pg g^-1^ FW from 20 d old hairy root cultures ^pK7^cal/rh*EPO*^HR^ with cal signal peptide (Fig 6B). The production of rhEPO^HR^ in this study is low as compared to the previous reports in tobacco plants [12], [16]. Though the expression level of rhEPO was high in young leaves of tobacco plants, the concentration of the recombinant protein gradually decreased in relative to TSP of the matured leaves [16]. Hence, the hairy root culture system established in this study for the expression and secretion of rhEPO^HR^ into the spent medium is highly advantageous due to its stable expression of rhEPO^HR^ and simplified downstream process of protein purification. The yield of rhEPO^HR^ can be enhanced by culturing the hairy root cultures in large scale fermenters by optimizing the growth parameters including the culture medium with minimal nuturients, temperature maintenance, low intensity light and gentle shaking (in case of liquid medium).

## Conclusion

The current study provides a new platform for the production of rhEPO^HR^. The addition of PVP into the culture medium enhanced the level of rhEPO^HR^ secreted from the hairy root culture ^pK7^cal/rhEPO^HR^ with cal signal peptide. We also report the regeneration of transgenic plantlets ^pK7^cal/rhEPO^RP^ from the hairy root culture with cal signal peptide.

## Abbreviations

ARPE: a retinal pigment epithelial cell line
BrdU: bromo-deoxyuridine
Caco-2: Caucasian colon adenocarcinoma cells
CaMV: cauliflower mosaic virus
MTT: 3-(4,5-dimethylthiazol-2-yl)-2,5-diphenyl tetrazolium bromide
rhEPO: recombinant human erythropoietin
